# Mapping the use of computational modelling and simulation in clinics: A survey

**DOI:** 10.3389/fmedt.2023.1125524

**Published:** 2023-04-17

**Authors:** Raphaëlle Lesage, Michiel Van Oudheusden, Silvia Schievano, Ine Van Hoyweghen, Liesbet Geris, Claudio Capelli

**Affiliations:** ^1^ Virtual Physiological Human Institute, Leuven, Belgium; ^2^ Centre for Sociological Research, Life Sciences and Society Lab, KU Leuven, Leuven, Belgium; ^3^ UCL Institute of Cardiovascular Science & Great Ormond Street Hospital for Children, London, United Kingdom; ^4^ Cardiorespiratory Unit, Great Ormond Street Hospital for Children NHS Foundation Trust, London, United Kingdom; ^5^ Prometheus, Division of Skeletal Tissue Engineering Leuven, KU Leuven, Leuven, Belgium; ^6^ Biomechanics Section, KU Leuven, Leuven, Belgium; ^7^ Biomechanics Research Unit, GIGA in Silico Medicine, University of Liège, Liège, Belgium

**Keywords:** in silico medicine, computer modelling, simulations, clinicians, translation, trust in technology, communities

## Abstract

*In silico* medicine describes the application of computational modelling and simulation (CM&S) to the study, diagnosis, treatment or prevention of a disease. Tremendous research advances have been achieved to facilitate the use of CM&S in clinical applications. Nevertheless, the uptake of CM&S in clinical practice is not always timely and accurately reflected in the literature. A clear view on the current awareness, actual usage and opinions from the clinicians is needed to identify barriers and opportunities for the future of *in silico* medicine. The aim of this study was capturing the state of CM&S in clinics by means of a survey toward the clinical community. Responses were collected online using the Virtual Physiological Human institute communication channels, engagement with clinical societies, hospitals and individual contacts, between 2020 and 2021. Statistical analyses were done with R. Participants (*n* = 163) responded from all over the world. Clinicians were mostly aged between 35 and 64 years-old, with heterogeneous levels of experience and areas of expertise (*i.e.,* 48% cardiology, 13% musculoskeletal, 8% general surgery, 5% paediatrics). The CM&S terms “Personalised medicine” and “Patient-specific modelling” were the most well-known within the respondents. “In silico clinical trials” and “Digital Twin” were the least known. The familiarity with different methods depended on the medical specialty. CM&S was used in clinics mostly to plan interventions. To date, the usage frequency is still scarce. A well-recognized benefit associated to CM&S is the increased trust in planning procedures. Overall, the recorded level of trust for CM&S is high and not proportional to awareness level. The main barriers appear to be access to computing resources, perception that CM&S is slow. Importantly, clinicians see a role for CM&S expertise in their team in the future. This survey offers a snapshot of the current situation of CM&S in clinics. Although the sample size and representativity could be increased, the results provide the community with actionable data to build a responsible strategy for accelerating a positive uptake of *in silico* medicine. New iterations and follow-up activities will track the evolution of responses over time and contribute to strengthen the engagement with the medical community.

## Introduction

Computational modelling and simulation (CM&S) involve the use of numerical tools to create virtual models of complex systems to better understand processes and predict effects of different interventions. CM&S can be used in medicine to mimic biological processes with the goal of understanding pathophysiology, improving diagnosis, treatment, or the prevention of diseases (1–5). Ever since the first numerical simulations, CM&S has been applied to model patient-specific conditions envisioning to become an aid of the decision-making process in complex clinical cases (6–12).

Whilst healthcare systems worldwide face increasing demand for reducing costs without compromising patient safety and outcomes, the translation of CM&S to clinics has been advocated as a support to improve the modern delivery of personalized healthcare (13, 14). Continuous advances in imaging technology, proliferation of data, improved simulation algorithms and steady rise of computational power increasingly contribute to CM&S used in clinics not only as decision support system but also to enrich the understanding of the physiological state and predict future states under different scenarios (15–18). Further, CM&S has emerged as the engine of *in silico* clinical trials where the outcomes of interventions can be predicted as based only on populations of virtual patients (19–21).

Despite the abundance of innovations in CM&S, the clinical uptake has not been consistent. Generally, the adoption of CM&S is still in its early stages. Recently published position papers and systematic reviews (22, 23) helped to identify general barriers to such adoption. These include both technical (validation, testing, integration) and legal & ethical challenges (regulation, costs and access). However, increased numbers of experiences have been reporting the use of *in silico* tools in clinics (24–27). This confirms a slow transformation which is likely to be happening in the real clinical world, but not necessarily captured timely by the literature.

Thus, this study aimed to investigate the status of uptake of CM&S in clinical practice by capturing the current clinicians' perspectives through a community survey. The questionnaire focused on taking a snapshot of the levels of knowledge and acceptance of CM&S within the medical community, and on their reported experiences with perceived benefits and barriers. The results of this study hopefully contribute to enriching the understanding of the clinical translation of CM&S.

## Context (setting and population)

The Virtual Physiological Human Institute (VPHi) reached out to medical stakeholders to record their experiences and opinions on the use of CM&S in clinical practice. In this study, CM&S were widely referred to any numerical methods or programming language used to model clinical context, either physics-based or data-driven. The questionnaire was designed by the authors of this paper, based on an extensive literature review, which helped to identify the following three critical elements to evaluate the use of CM&S in healthcare settings: knowledge, experience and opinions. The questionnaire was structured in two sections to capture:

1. Demographic data of the respondents: medical specialty, country of work, clinical position, age group, any academic and research experience (*i.e.,* number of articles published; research grants; collaborations; involvement in trials)
2. Respondents' awareness and familiarity with CM&S methodologies: examples of applications to their clinical practice, presence of team members dedicated to CM&S in clinical settings, level of confidence, level of trust, type of use, medical field, number of clinical cases and examples of applications of CM&S over the past 12 months.

In addition, to gain a better understanding of the perception of in silico technologies and CM&S by clinicians, their opinions were recorded with respect to trust in CM&S, accuracy, efficacy, accessibility, credibility, as well as perceived benefit for their practice. Responses were analysed both globally and between groups of previous users and nonusers to evaluate if alternative tendencies were observed based on the previous experiences of the respondents with CM&S.

The full text of the questionnaire is available via Zenodo (https://doi.org/10.5281/zenodo.7704367). Data were collected between November 2020 and March 2021 through the online platform SurveyMonkey (Momentive Inc, USA). Participation to the survey was open to active medical doctors, and dissemination was achieved via the following channels:

• VPHi communications: regular announcements on the newsletter, VPH website (www.vph-institute.org), posts on social media, announcements during regular webinars;
• Institutional clinical partners: Great Ormond Street Hospital (London, UK), University Hospital Leuven (UZ Leuven, Leuven, Belgium) and University Hospital Liège (CHU Liège, Liège, Belgium), Necker–Enfants Malades Hospital (Paris, France), Ospedale Pediatrico Meyer (Florence, Italy), and Nelson Mandela Children Hospital (Johannesburg, South Africa).
• Engagement with professional societies: European Society of Biomechanics, European Society of Cardiology, Association of European Paediatric and Congenital Cardiology, and Congenital & Structural Intervention;
• Word of mouth through individual members of the VPH Institute.

Statistical analyses were performed using R Statistical Software (v.3.5.1; R Core Team; R: A language and environment for statistical computing. R Foundation for Statistical Computing, Vienna, Austria. URL https://www.R-project.org/.). The categorical data were nominal and ordinal. Associations between categorical data were investigated with the Chi test of independence, or the test Fisher exact test of independence when the number of observations in one category was too low (independence rejected for *p* < 0.05). Cochrane-Armitage test of association (two sided) was used for those cases where the nominal variable was a binary factor and the other variable was ordered. Unpaired non-parametric Wilcoxon test was used for comparing the level of trust between two different groups. Missing values (NA) were ignored for the data analyses; the number of answers counted for each analysis is reported in the corresponding figure captions.

A population stratification analysis was carried out. The respondents were grouped into CM&S users and nonusers to evaluate if alternative tendencies were observed based on respondents' previous experiences with CM&S. This filtering was done based on survey question #4, which asked respondents to list all CM&S methods they had already applied to their practice. Respondents who had selected “None” were considered as “nonusers”, while all others were counted as “users”.

The questionnaire, the collected data and the scripts to analyse them are archived by the VPHi community in Zenodo (https://zenodo.org/communities/vph-institute). In particular, the questionnaire and the raw dataset collected for this study (csv file) are accessible via: https://doi.org/10.5281/zenodo.7704367. The code to analyse the data is accessible via https://doi.org/10.5281/zenodo.7704491.

## Results

A total of 163 surveys were collected and analysed during the period of this study. The response rate was 83%. The average completion time was 8m:02s ± 4m:06s.

Demographics of participants to the survey are reported in Table 1 as regards to age (about 30% of the respondents from the group 35–44 years old), medical area of expertise (40% from cardiac), clinical (22% head of units) and academic (15% professor) positions held, number of publications and grants, and countries of work. The majority of respondents (86%) had collaborations in Europe; 21% in North America; 7% in Africa, 7% in Asia, 3% in South America and 1% in Oceania. With regards to experience in trials and regulatory pathway, about 48% of respondents declared that they have participated in clinical trials, whilst nearly 27% of them has been involved in the submission process for pharma or device approvals to regulatry bodies.

**Table 1:** Demographic characteristics of the survey respondents (*N* = 163).

	*n*	%
**Age**		
25–34	14	8.7
35–44	49	30.1
45–54	46	28.2
55–64	40	24.5
>65	11	6.7
n/a	3	1.8
**Medical areas**		
Cardiac	65	39.9
Musculoskeletal	17	10.4
General surgery	11	6.7
Paediatrics	7	4.3
Rheumatology	7	4.3
Imaging	6	3.7
Oncology	5	3.1
Anaesthetics	4	2.5
Other	13	8.0
n/a	28	17.2
**Current clinical position (*n *= 105)**		
Head of unit	36	22.1
Consultant	28	17.2
Registrar/MD	23	14.1
Fellow/Junior Doctors	6	3.7
Surgeon	5	3.1
Other	7	4.3
**Academic post (*n *= 46)**		
Professor	25	15.3
Associate professor	9	5.5
Researchers	12	7.4
**Publications**		
<10	39	
11–100	54	
>100	34	
**Grants in CM&S**		
None	56	
<1M Euros	15	
1–5M	18	
>5M	9	
**Countries**		
Belgium	25	15.3
Italy	25	15.3
United Kingdom	25	15.3
United states	13	8.0
France	11	6.7
Germany	11	6.7
Spain	10	6.1
South Africa	6	3.7
The Netherlands	5	3.1
Egypt	3	1.8
Other	18	14.1

*n* represents the number of response (counts) while the column (%) reports the percentage with respect to the total number of answers.

### Knowledge of CM&S in clinics

**Figure 1:**
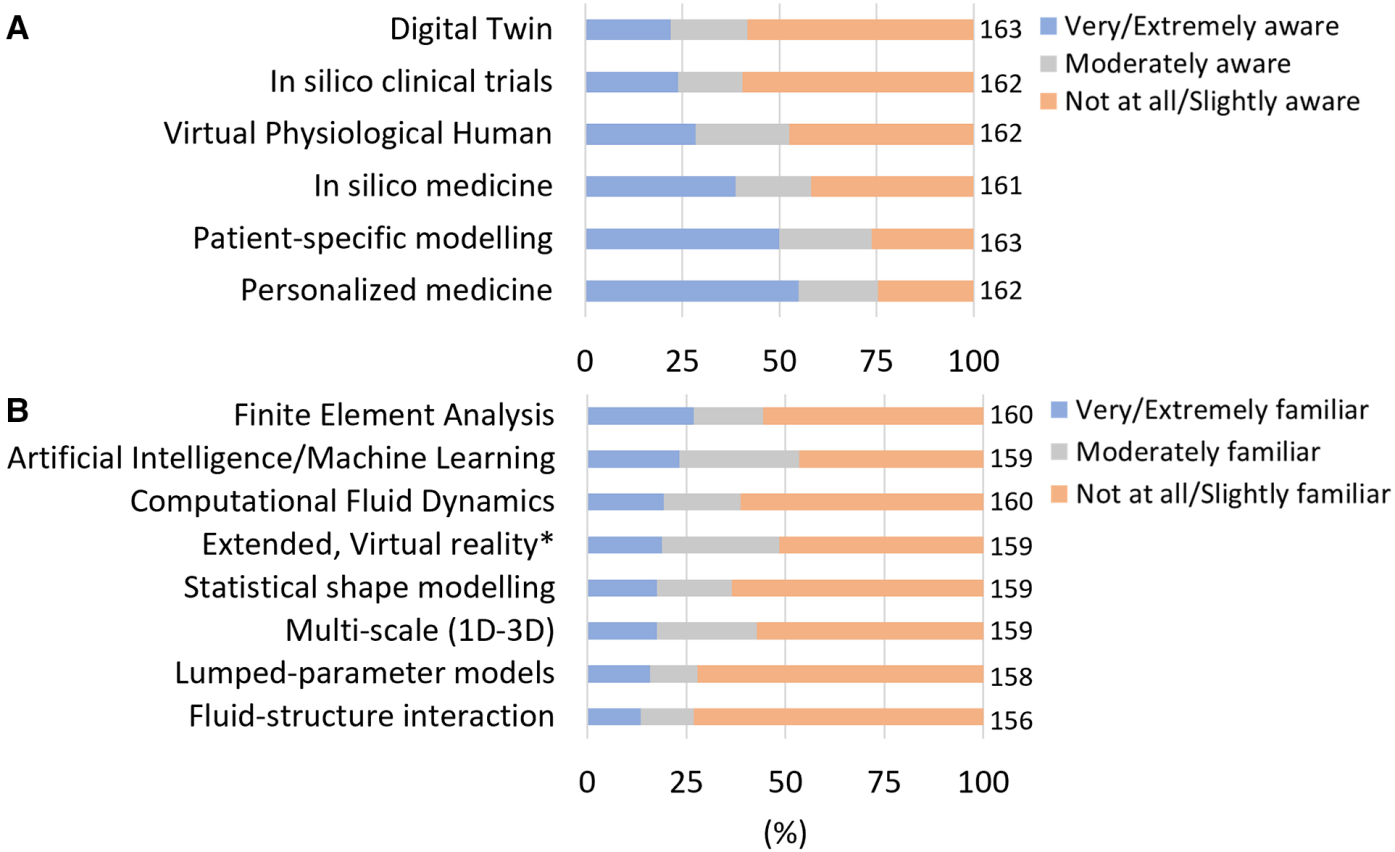
Awareness and familiarity with *in silico* terms and methodologies. (**A**) Level of awareness in in silico terms. Respondents who answered very and extremely aware (resp. Not at all aware and slightly aware) were grouped. (**B**) Level of familiarity with computational methods related to in silico technologies. Respondents who answered very and extremely familiar (resp. Not at all aware and slightly familiar) were grouped. The number of responses (*n*) amounting to 100% of the responses is reported on the right of each bar.

The levels of awareness and familiarity to different general in silico terms and technical tools is reported in Figures 1A,B, respectively. “Personalised medicine” and “Patient-specific modelling” were the most known terms (55% and 50% of respondents answered to be very or extremely aware of the respective terminology). “In silico clinical trials” and “Digital Twin” were the least known terms (60% and 58% were not at all/slightly aware, respectively), among our sampled group. Overall, the level of awareness for the terms “virtual physiological human” and “in silico medicine” was moderate. Respondents appeared not to be familiar with technical tools as in average, only 19.1% + - 4.0 of respondents declared familiarity with the different methods. The most known methods were Finite Element Analysis (∼27%); AI (∼23%) and Computational Fluid Dynamic (∼19%).

### Experience of CM&S in clinics

**Figure 2:**
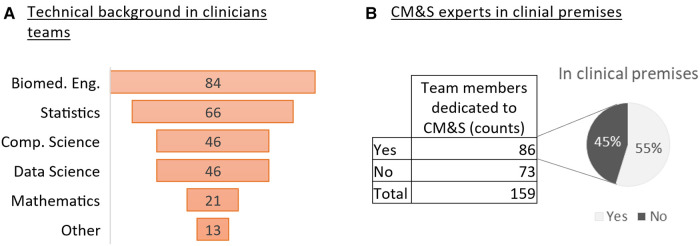
CM&S and technical expertise in clinical teams (**A**) Ranking of CM&S related backgrounds from the most represented to the less in the clinicians’ teams (survey question #2) (**B**) Number of respondents who declares having team members dedicated to CM&S and percentage of them being based in clinical premises.

Clinical teams appear to be open to a multidisciplinary approach to medicine, which includes also CM&S expertise. Most respondents (70%) have experts in CM&S in their team distributed between biomedical engineering (30% of the respondents); statistics (24%), computer science (17%) and data science (17%) (Figure 2A). Other recorded types of expertise included database development, computational biophysics, pharmaco-informatics, nuclear and mechanical engineering. The proximity between collaborators of different disciplines appears to be very common among the respondents who have CM&S expertise in their team (Figure 2B).

**Figure 3:**
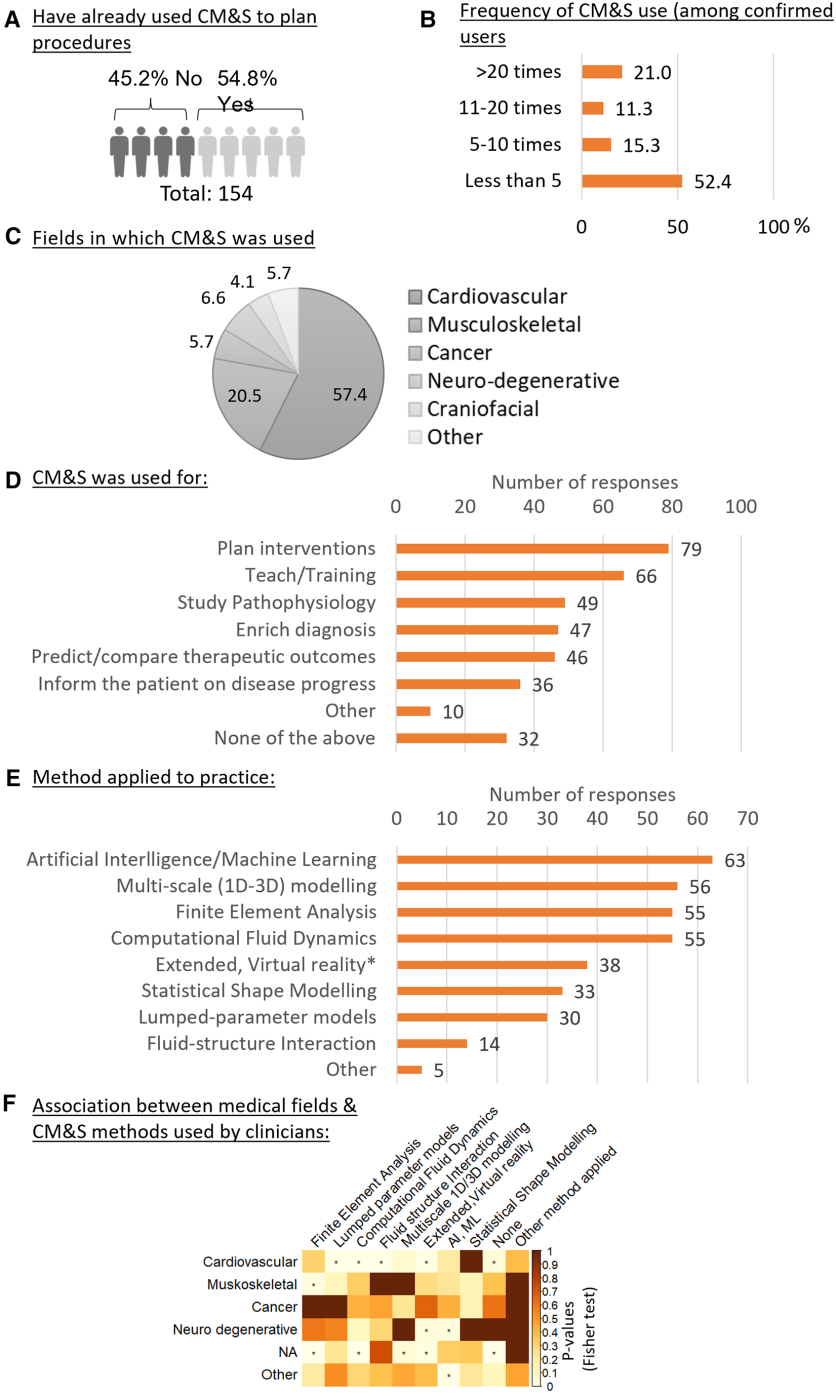
Usage of CM&S and related methods in the clinics. (**A**) percentage of clinicians who have already used CM&S for planning procedures (question #10). (**B**) Frequency of usage of CM&S among clinicians who have already used it to plan procedure. (**C**) Fields in which CM&S was used to plan intervention, expressed in percentage of *n* = 122 responses. (question #11) (**D**) Purpose of application of CM&S by clinicians ranked based on number of responses (question #9) (**E**) Ranking of CM&S related methods from the less to the most applied to clinical practice by respondents (question #4). (**F**) Association between the medical field in which respondents have applied CM&S (questions #11) and CM&S method that have been applied by the respondents. Test of independence: Fisher Exact test, the independence is rejected for *p*-values < 0,05, indicated with (*).

Besides technical expertise and awareness, CM&S were used to plan procedures by 55.0% of the respondents (Figure 3A). The majority of them (52%) did so less than 5 times in the year prior to the survey, 26% between 5 and 20 times, and 21.5% more than 20 times (Figure 3B). The level of usage varied between all medical fields. Among the respondents who had used CM&S to plan intervention, “Cardiovascular” was the most represented field (57%), followed by “Musculoskeletal” (21%), “Neurodegenerative diseases” (7.%) and “Oncology” (6%) (Figure 3C).

The investigation of other types of activities for which CM&S was generally used highlighted how “Teaching” was the most frequent application (53%), followed by “Study Pathophysiology” (39%), “Enrich diagnosis” (38%), “Predict/compare different therapeutic outcomes” (36%), and “Inform the patient on the disease progression” (29%) (Figure 3D). In terms of numerical tools, Artificial Intelligence/Machine Learning was reported to be most applied (*n* = 63), followed by multi-scale modelling (*n* = 56), Finite Element Analysis and Computational Fluid Dynamics (*n* = 55) (Figure 3E).

By looking at the statistical dependency (associations) between the field in which the clinicians had used CM&S to plan intervention and their usage of a certain *in silico* method (Figure 3F), a positive association was found between the cardiovascular field and the usage of (1) lumped parameter models, (2) computational fluid dynamics, (3) fluid interaction and (4) extended & virtual reality methods. This was not the case for other medical fields, except for the extended & virtual reality that was also positively associated, along with AI and machine learning, with the neurodegenerative field, although the sample size for the neurodegenerative clinicians who had used CM&S was small. Finally, the musculoskeletal field was positively associated with Finite Element Analysis, as could be expected given the importance of (bio)mechanics in this field.

### Opinions on the use of CM&S in clinics

**Figure 4:**
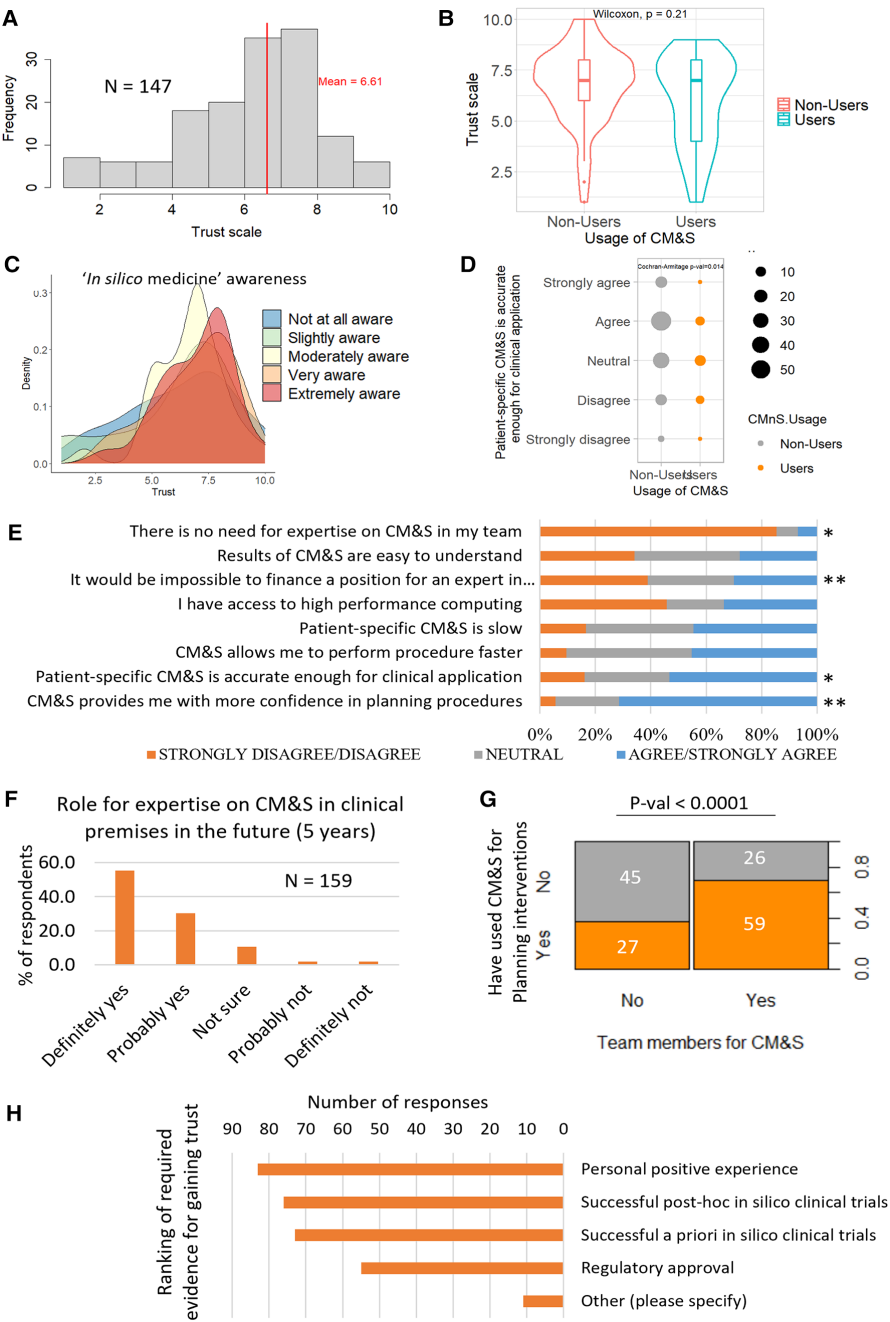
(**A**) Overall trust level distribution for in silico technologies, *n* = 147 (**B**) Comparison of trust between CM&S users and nonusers. The grouping between user and nonuser was done according to the responses to question #4 of the survey (see Method). The non-parametric Wilcoxon test indicates that there is no significant difference of trust between users and nonusers. (*n* = 147) (**C**) Distribution of trust, grouped by level of awareness in “in silico medicine” concepts, *n* = 147 (**D**) Comparison of agreement with statement on CM&S accuracy between users and nonusers showed that the level of agreement was dependent on the group. *P*-value is displayed for the Cochran-Armitage test of association to compare ordinal variable between two groups, *N* = 156 (**E**) Level of agreement with various statements related to CM&S (*N* = 156–158 depending on the statement). The difference of answers between the user groups were evaluated with Cochrane Armitage test: * and ** indicate *p*-values < 0.05 and <0.005 respectively. Detailed *p*-values in Supplementary Figure S2. (**F**) Percentage of respondents seeing a role for expertise in CM&S in their team in the next 5 years (**G**) Association between the usage of CM&S to plan intervention and the presence of dedicated members in the team. (**H**) Type of evidence requested by clinicians to trust CM&S, ranked by number of votes (multiple choice possible).

Clinicians rated their level of trust in CM&S (Figure 4A) with a 6.61 ± 1.97 out of 10 on average. The level of trust amongst respondents followed a right-skewed distribution. No trust difference was observed between respondents who had already applied CM&S methods and respondents who never did (Figure 4B). Trust was generally higher amongst those respondents who were very and extremely aware of *in silico* concepts (*i.e.*, *in silico* medicine, patient-specific modelling, *in silico* clinical trials, Virtual Physiological Human institute, Personalised medicine, Digital Twin) (Figure 4C, Supplementary Figure S1). However, respondents with a lower level of awareness for those concepts (slightly or not at all aware) still rated >5.5 out 10 their level of trust, on average. Moreover, within some groups with the same awareness level, bimodal distributions for the level of trust were observed, suggesting non-homogeneous opinions (Figure 4C, Supplementary Figure S1). These results also indicate that there is no strict relation between level of trust and level of awareness regarding *in silico* technologies, pointing to further tracks of investigation (see discussion).

In terms of accuracy, we observed that patient-specific CM&S was considered accurate enough for clinical applications for most respondents: 53.2% agreed or strongly agreed, although about 16% of respondents disagreed. Nonusers appeared to consider CM&S accurate more than users (Cochrane Armitage test *p*-value = 0.014) (Figures 4D,E). Supplementary Figure S2 reports the *p*-values for the difference of trends in opinion between users and nonusers for the statements listed in Figure 4E.

More than 70% of the respondents agreed or strongly agreed with the statement “Computer modelling and simulation provides me with more confidence in planning procedure” (158 respondents) (Figure 4E). Slightly less respondents agreed with the fact that CM&S allowed them to perform procedures faster, although <10% disagreed (45% neutral) (Figure 4E). A bit less than half of the respondents perceived patient-specific CM&S as slow (Figure 4E). No significantly different opinions were recorded in this field between users and non-users of CM&S (Figure 4E).

In terms of complexity of *in silico* technologies, the opinions were split equally with almost ⅓ of respondents who found CM&S results easy to understand, ⅓ who found them difficult to understand and the last third being neutral (Figure 4E).

Regarding skills and expertise, >85% of respondents foresee a role for expertise on CM&S in their team in the next 5 years (Figure 4F); this propensity was positively associated with the pre-existence of members dedicated to CM&S in their team (Supplementary Figure S3). We also observed a significant positive association between the presence of team members dedicated to CM&S and the fact they had already used CM&S to plan interventions (Figure 4G), suggesting that clinical teams who have members dedicated to CM&S are more likely to use those technologies in their clinical practice. Nevertheless, 30% of respondents affirm it would be impossible to finance a position for an expert in computer modelling and simulation in their organisation (Figure 4E). A potential barrier when it comes to practical issues, could be access to high performance computing. More than 45% of respondents do not have access to such a facility, although a large part of respondents may simply not be aware of such resources (20% of neutral responses) (Figure 4E).

Finally, in terms of generation of evidence, a positive personal experience, successful post-hoc *in silico* trials and successful *a priori in silico* trials were ranked 1st, 2nd and 3rd respectively (with similar amounts of preferences) when asked which kind of evidence they would require to trust the outcome of CM&S. Regulatory approval came after, with about ⅕ of the voters who selected that option (Figure 4H). Other opinions that were spontaneously proposed by respondents included dedicated research studies, such as randomised clinical trials, more involvement of clinicians from the initiation of modelling projects—“it would be better to invite clinicians already from the beginning”-, validation methods with clinical data—“validation of pre-operative CFD with post-op MRI”—or user-friendly interface facilitating data processing and outcome interpretation for decision making—“easy to use interface and fast method from data to information [..]”.

## Discussion

This report presents the findings of a study aimed to capture spreading, level of acceptance and barriers that clinicians see related to the use of computational models and simulations in their practice. Data were collected exclusively among the clinical community via a dedicated survey. The main objective of such survey was recording real world experiences and opinions from the clinical stakeholders, not usually documented in CM&S research publications. To the best of our knowledge, this is the first investigation of this kind and can represent a benchmark for future comparisons.

Our findings show how terms related to CM&S are generally not stranger to the clinical community. However, there is discrepancy among the different expressions which are commonly used by the modelling community. In particular, terms such as “Digital twin” and “*In silico*” appear not to be particularly familiar to most clinicians, suggesting that more effort should be dedicated to efficient communication. On the other side, “Personalized Medicine” was found to be the most well-known expression. This might be due to the fact that this term is already widely used by clinicians, especially in the field of genetics. In future analysis, it will be useful to monitor the evolving familiarity with such glossary. According to the collected data, CM&S have been used mainly to support decisions related to the planning of procedures and for teaching activities. Whilst planning is consistent with what reported in the literature on translation of CM&S towards clinics, such extensive use for teaching activities is not properly represented and deserves follow-up and more granular future analyses. Cardiovascular and musculoskeletal are the areas where CM&S have found their highest usage to date. This finding is in accordance with the evolution of CM&S in such applications as reported in the literature of the past couple of decades. With regards to the methods which have been applied most frequently, there is a surge of AI/ML techniques followed by physics-based modelling techniques, even though the latter methodologies have been around for much longer. Although the overall use of CM&S in clinics is not routine yet, it was recorded how clinicians generally trust CM&S; however, the concept of trust should be investigated further for example by looking at reliability confidence. Importantly, such opinion on trust is consistent across groups with different familiarity towards such methods. It is crucial for the community of modellers to build on such credit and provide more evidence on the impact that CM&S can have in improving healthcare delivery.

Collection of the respondents' opinions allowed exploratory identification of strengths, weaknesses, opportunities, and threats (SWOT) related to the use of CM&S in clinics. The following proposed SWOT analysis could be used to develop a strategy to successfully translate in silico technologies to healthcare.

• Strengths: an overall awareness of the respondents towards computational terminology; high confidence in the positive role played by CM&S in planning procedures; sufficient accuracy of the numerical tools to provide patient-specific results; a relatively high level of trust in CM&S; and, lastly, an overall confidence on the rising role envisioned for CM&S in clinical routine. The latter was found to be positively associated with the pre-existence of team members dedicated to CM&S; this seems to confirm the positive effect of a close collaboration between technical experts and clinicians on the ground.
• Weaknesses: difficulties in accessing technical expertise and computing resources; perceived slow turnaround time of simulations' results; and the perceived limitation of CM&S applications to a few medical areas.
• Opportunities: confidence in growth of methods, data and experiences in clinics; prospect of exploring applications in teaching and training; requirement for clinical validation of CM&S tools; positive attitudes to contribute to the early development of CM&S, and the natural rise of new generations of healthcare providers who will be increasingly keen to use digital tools.
• Threats: lack of a shared pathway to gather evidence to support a safe use of *in silico* technologies in clinics; potential issues in scaling up the adoptions of CM&S as suggested by the scarce frequency of applications; low confidence of clinicians regarding regulatory accreditations as compared to personal experience; lack of funding to recruit CM&S expertise within clinical premises; and, a low level of awareness of specific terms (e.g., “Digital twin”) which might lead to confused communication between stakeholders.

There are some limitations associated with this study. First, a rather general, exploratory approach has been adopted in designing the survey, as this is the first time a survey of this kind has been proposed to the clinical community. While this approach has helped identifying priorities and sensitising the community to emerging issues (many of which will develop along the way), future surveys can be modulated to capture more specific experiences and opinions on the adoption of CM&S in clinics. Second, the respondents were recruited mainly by exploring and extending the network of contacts of the VPH community which is an advocate of the development of CM&S in healthcare. Such sample bias and sample size might influence the generalization of the findings. Third, there was not an equal distribution among different medical fields, geographical areas, or among different demographic groups. A low statistical power for some specific subgroups (e.g., neurodegenerative field) might have had an impact on the identifications of specific associations with the adoption of different CM&S techniques. Therefore, broadening the profiles of the respondents will be needed in future analyses. In addition, ongoing projects such as Sano (https://sano.science/) and InSilicoWorld (https://insilico.world/), will contribute putting those findings in perspective with inclusion of different stakeholders. Finally, it must be kept in mind that the presented data were collected in 2021. Within a field which is evolving extremely rapidly, the latest changes and trends might not have been fully captured. In particular, the community has seen an increasing interest to concepts such as “digital twin” or “in silico trials”. Hence, longitudinal analyses will be performed to evaluate how this is evolving and changing the impact within the clinical community as well.

In conclusion, this study reports the findings of a direct investigation within the clinical community about the adoption and perception of CM&S in clinics. Understanding the perspective of healthcare end-users is crucial to guide the adoption of *in silico* medicine for increasing patients' benefit. The collected data show an overall readiness of the clinical community for increased adoptions of CM&S technologies. Future studies will need to track the evolution of such experience and opinions over time.

## Data Availability

The questionnaire, the collected data and the scripts to analyse them are archived by the VPHi community in Zenodo (https://zenodo.org/communities/vph-institute). In particular, the questionnaire and the raw dataset collected for this study (csv file) are accessible via: https://doi.org/10.5281/zenodo.7704367. The code to analyse the data is accessible via https://doi.org/10.5281/zenodo.7704491.
