# Supplementary figures and images for: Mapping the use of computational modelling and simulation in clinics: A survey
Source: Front Med Technol. 2023 Apr 17;5:1125524. doi: 10.3389/fmedt.2023.1125524 (PMC10150234; doi:10.3389/fmedt.2023.1125524)

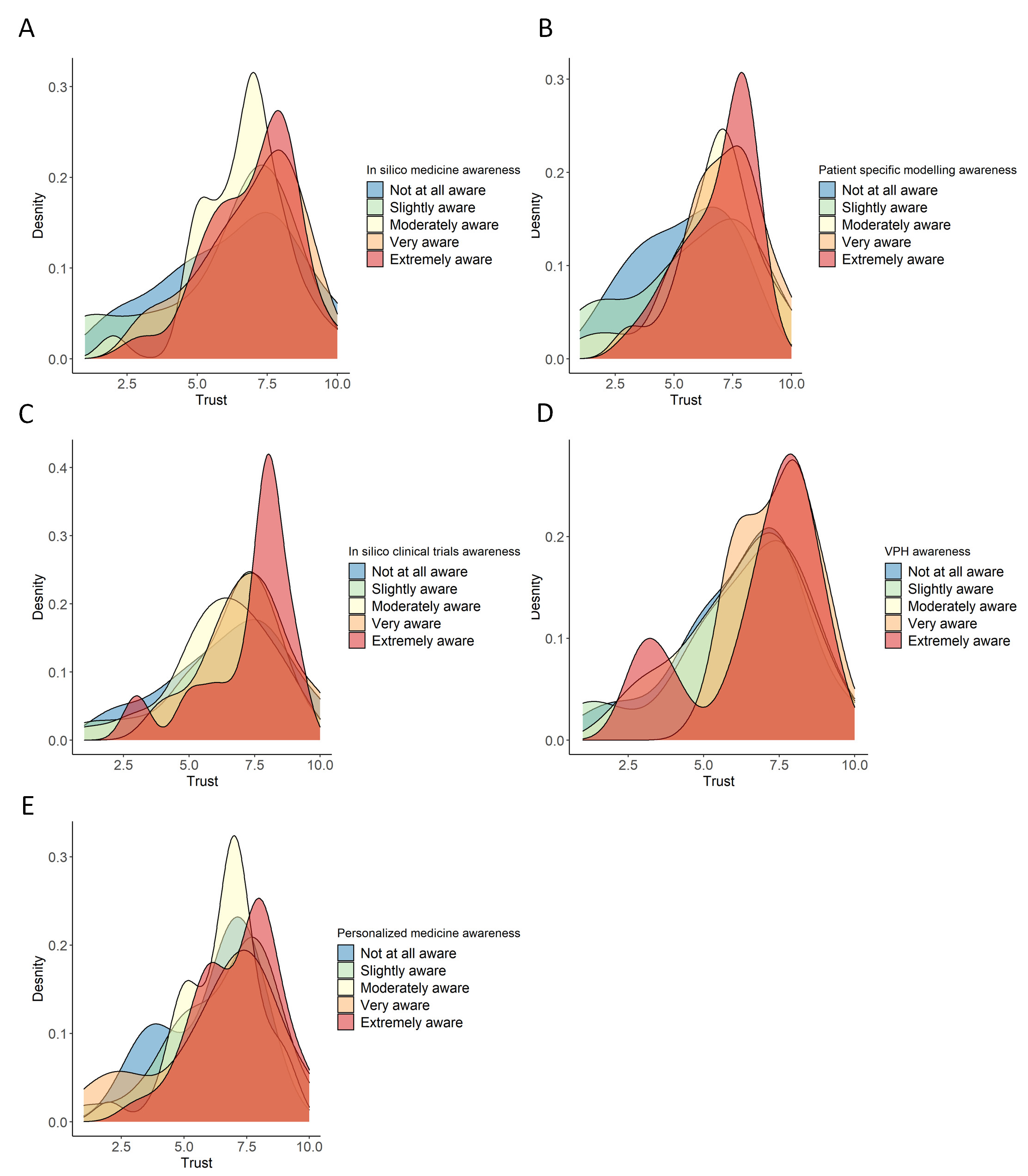

Supplement: Supplementary file 1 [file Image1.jpg]

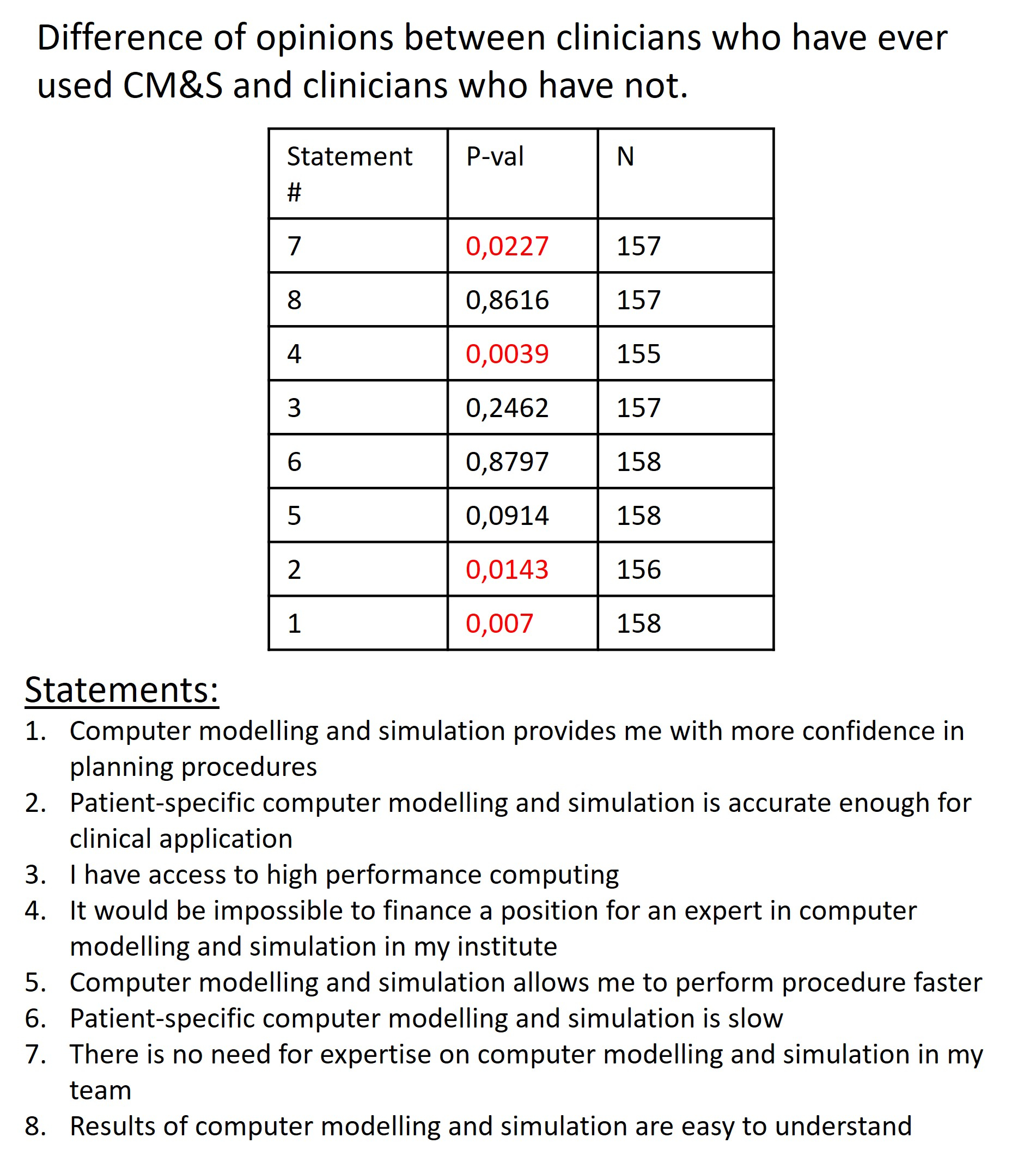

Supplement: Supplementary file 2 [file Image2.jpg]

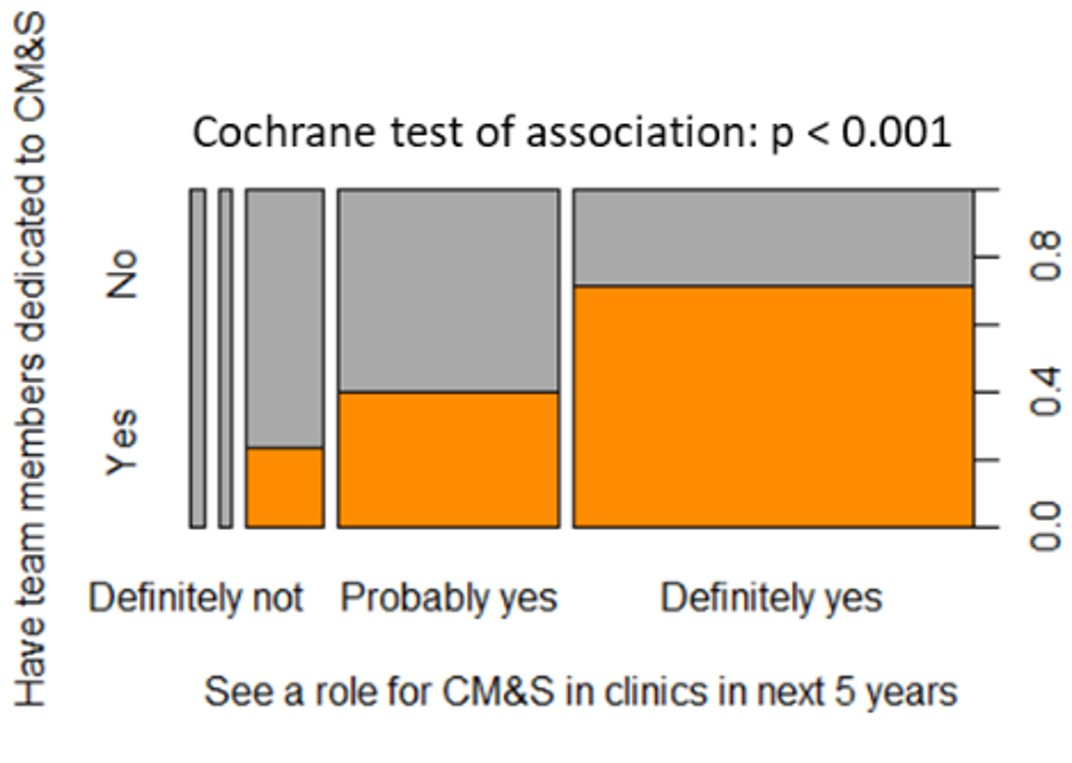

Supplement: Supplementary file 3 [file Image3.jpg]
